# Global DNA Methylation in the Chestnut Blight Fungus *Cryphonectria parasitica* and Genome-Wide Changes in DNA Methylation Accompanied with Sectorization

**DOI:** 10.3389/fpls.2018.00103

**Published:** 2018-02-02

**Authors:** Kum-Kang So, Yo-Han Ko, Jeesun Chun, Jyotiranjan Bal, Junhyun Jeon, Jung-Mi Kim, Jaeyoung Choi, Yong-Hwan Lee, Jin Hoe Huh, Dae-Hyuk Kim

**Affiliations:** ^1^Institute for Molecular Biology and Genetics, Center for Fungal Pathogenesis, Chonbuk National University, Jeonju, South Korea; ^2^Department of Biotechnology, College of Life and Applied Sciences, Yeungnam University, Gyeongsan, South Korea; ^3^Department of Bio-Environmental Chemistry, Wonkwang University, Iksan, South Korea; ^4^Department of Agricultural Biotechnology, Seoul National University, Seoul, South Korea; ^5^Department of Plant Science, Seoul National University, Seoul, South Korea

**Keywords:** DNA methylation, whole genome bisulfite sequencing, genome-wide association study, mitogen-activated protein kinase kinase kinase, sectorization, epigenetics

## Abstract

Mutation in *CpBck1*, an ortholog of the cell wall integrity mitogen-activated protein kinase kinase kinase (MAPKKK) of *Saccharomyces cerevisiae*, in the chestnut blight fungus *Cryphonectria parasitica* resulted in a sporadic sectorization as culture proceeded. The progeny from the sectored area maintained the characteristics of the sector, showing a massive morphogenetic change, including robust mycelial growth without differentiation. Epigenetic changes were investigated as the genetic mechanism underlying this sectorization. Quantification of DNA methylation and whole-genome bisulfite sequencing revealed genome-wide DNA methylation of the wild-type at each nucleotide level and changes in DNA methylation of the sectored progeny. Compared to the wild-type, the sectored progeny exhibited marked genome-wide DNA hypomethylation but increased methylation sites. Expression analysis of two DNA methyltransferases, including two representative types of DNA methyltransferase (DNMTase), demonstrated that both were significantly down-regulated in the sectored progeny. However, functional analysis using mutant phenotypes of corresponding DNMTases demonstrated that a mutant of *CpDmt1*, an ortholog of RID of *Neurospora crassa*, resulted in the sectored phenotype but the *CpDmt2* mutant did not, suggesting that the genetic basis of fungal sectorization is more complex. The present study revealed that a mutation in a signaling pathway component resulted in sectorization accompanied with changes in genome-wide DNA methylation, which suggests that this signal transduction pathway is important for epigenetic control of sectorization via regulation of genes involved in DNA methylation.

## Introduction

*Cryphonectria parasitica* (Murrill) Barr, the causal agent of chestnut blight, destroyed the chestnut forests in North America at the beginning of the 20th century (Van Alfen, 1982). However, strains containing Cryphonectria Hypovirus 1 (CHV1) exhibit characteristic symptoms of lowered virulence, a phenomenon referred to as hypovirulence (Van Alfen et al., 1975; Anagnostakis, 1982; Nuss, 1992), and diverse hypovirulence-associated phenotypic changes such as reduced sporulation, female fertility, pigmentation, laccase production, and oxalate accumulation (Havir and Anagnostakis, 1983; Elliston, 1985; Rigling et al., 1989). The interaction between *C. parasitica* and hypovirus has been known for many years as a model system for the study of fungus–mycovirus interaction. In *C. parasitica*, several genes in the mitogen-activated protein kinase (MAPK) signaling pathway and its downstream targets are manipulated by the presence of hypovirus or are involved in pathogenicity (Park et al., 2004, 2012; Choi et al., 2005; Turina et al., 2006; Deng et al., 2007; Sun et al., 2009). Among these, studies on *CpBck1*, an ortholog of yeast Bck1 [a cell wall integrity (CWI) MAPKKK], have been conducted because a *CpBck1*-null mutant showed an unexpected phenotypic change involving sporadic sectorization (Lee and Levin, 1992; Kim et al., 2016). In addition, recent studies on *CpSlt2*, an ortholog of yeast Slt2 (a CWI MAPK), demonstrated that mutation in the *CpSlt2* gene resulted in the sectorization phenotype although the phenotype was not as severe as that of the *CpBck1* mutant (So et al., 2017). These results suggest that sectorization is the result of genetic changes in the CWI MAPK signaling pathway. Sectorization, defined as robust mycelial growth without differentiation, is common in many fungi; however, very little is known regarding its underlying genetic mechanism.

Recent studies on fungal gene regulation have revealed a novel aspect of a higher-hierarchical level of regulation (i.e., the roles of chromatin structure and modification, DNA methylation, and non-coding RNAs). This epigenetic machinery not only regulates the expression of individual genes but also plays an important role in global gene expression patterns (Strauss and Reyes-Dominguez, 2011). Methylation of selected cytosines in DNA is a prototypical epigenetic process that occurs in many eukaryotes and has been implicated in a variety of processes, including embryonic development, X-chromosome inactivation, genome imprinting, silencing of transposons, and gene regulation(Miura et al., 2001; Reik and Walter, 2001; Reik et al., 2001; Heard and Disteche, 2006; Weber and Schübeler, 2007). In fungi, DNA methylation exists from undetectable levels (≤0.1% of cytosine residues; Liu et al., 2012) to low but detectable levels (0.2–4.3% of cytosine residues; Storck et al., 1971; Antequera et al., 1984; Foss et al., 1993; Butler et al., 2009; Jeon et al., 2015) to markedly high levels (10–30% of cytosine residues; Montanini et al., 2014). Additionally, the methylated sites are generally clustered away from essentially unmethylated regions. Although the importance of DNA methylation in fungi is still unclear, *Neurospora crassa* and *Ascobolus immersus* are two well-known examples of DNA methylation that play roles in genome defense. Recent studies on the genome-wide methylation analysis indicated that DNA methylation occurs in and around genes, and fungal epigenetic entity contributed to fungal development as well as genome defense (Zemach et al., 2010; Jeon et al., 2015). Moreover, although some functions of DNA methylation have been identified, its regulation is not well understood.

In this study, we performed whole-genome bisulfite sequencing (WGBS) to examine the genome-wide distribution of DNA methylation in *C. parasitica* and to determine the implications of DNA methylation in sectorization in the mutant of *CpBck*1 from *C. parasitica*. Our results revealed genome-wide patterns of DNA methylation and showed massive changes in DNA methylation in sectored progeny of the *CpBck*1 mutant, suggesting that a specific MAPK signaling pathway plays a role in DNA methylation, resulting in sporadic occurrence of sectors in which DNA methylation differed significantly from that of the parental wild-type.

## Materials and Methods

### Fungal Strains and Growth

The *C. parasitica* strain EP155/2 (ATCC 38755), used as the wild-type strain, and its isogenic hypovirus-CHV1-713-containing strain, UEP1, were cultured on PDAmb plates under constant low light at 25°C (Kim et al., 1995). The culture conditions and methods for preparation of the primary inoculum for liquid culture were described previously (Kim et al., 1995). The collected mycelia were lyophilized and stored at -70°C until use (Powell and Van Alfen, 1987). Strains used for the analysis of DNA methylation were the *CpBck1*-null mutant (TdBCK1), the sectored progeny of the *CpBck1*-null mutant (TdBCK1-S1), and the complemented strain of the sectored *CpBck1*-null mutant strains (TcBCK1-S1) from our previous study (Kim et al., 2016). The wild-type EP155/2 strain was included for comparison purposes.

### Methylated DNA Quantification

Genomic DNA was extracted using lyophilized mycelia (Kim et al., 1995) and was further purified using a G25 column (GE Healthcare, Little Chalfont, BUX, United Kingdom; Gobbi et al., 1990;Asanuma et al., 2013). Quantification of 5-methylcytosine (5mC) was performed using a MethylFlash^TM^Methylated DNA Quantification Kit including ME4 as a control (50% of 5-methylcytosine; Epigentek, New York, NY, United States), as described previously (Simmons et al., 2013).

### Whole-Genome Bisulfite Sequencing (WGBS)

Whole-genome bisulfite sequencing was performed as described previously (Kulis et al., 2012). Briefly, genomic DNA (5 μg) was mechanically sheared into fragments, and the fragments were end-repaired for ligation to a methylated adapter with a single-base ‘T’ overhang using Truseq DNA library preparation kit (Illumina, San Diego, CA, United States). The ligated products were size-selected by agarose gel electrophoresis, and bisulfite conversion of the fragments was performed as described previously using an EpiTect Bisulfite Kit (Qiagen, Manchester, MCH, United Kingdom). The treated product was amplified by PCR using a uracil-tolerant proofreading enzyme (Pfu Turbo Cx, Stratagene, La Jolla, CA, United States). Libraries were paired-end sequenced using the Illumina HiSeq2000 platform. Raw sequencing data were checked for quality using the FastQC program, and alignment and mapping with the reference genome data of *C. parasitica*^1^ were performed to identify the methylation of cytosine residues using Bismark and Bowtie2 software, as described previously (Xiang et al., 2010). The degree of DNA methylation was indicated by the methylation level and density, which were defined as the number of mC reads divided by the number of total reads covering the site and as the number of mC sites per 10 kb, respectively.

### Validation of WGBS by Bisulfite-PCR

Genomic DNA of *C. parasitica* was fragmented using restriction enzymes that cut outside target regions of interest and was subjected to manual bisulfite conversion (Colella et al., 2003; Clark et al., 2006). Based on the WGBS data, the target regions, which read count was more than 100 and methylation occurred at all context in all four samples, were randomly selected and analyzed. Amplified products were cloned using the pGEM-T Easy vector (Promega, Madison, WI, United States), and at least 18 independent clones were sequenced to analyze the target region. Primer pairs for bisulfite-PCR validation are shown in Supplementary Table S1.

### Cloning and Characterization of C5-DNA-Methyltransferase Genes, *CpDmt1* and *CpDmt2*

The genome database of *C. parasitica* was screened for C5-DNA-methyltransferase genes. Two putative C5-DNA-methyltransferase genes *CpDmt1* and *CpDmt2* were selected for further analysis. PCR amplification of *CpDmt1* was performed using the primers CPDMTC5_2549_F1 and CPDMTC5_2549_R2 and that of *CpDmt2* was performed using the primers CPDMTC5_1891_F1 and CPDMTC5_1891_R2 (Supplementary Table S1). The resulting 5.6-kb PCR amplicon of *CpDmt1* and 7.3-kb amplicon of *CpDmt2* were cloned separately into the pGEM-T Easy vector and sequenced using the dideoxynucleotide method with universal and synthetic oligonucleotide primers.

To obtain the cDNA clones of *CpDmt1* and *CpDmt2*, PCR was performed using reverse transcriptase (RT-PCR) with primers CpDmt1-mF1/CpDmt-mR1 and CpDmt2-mF1/CpDmt2-mR1 at nucleotide positions (nt) -8 to 14/3,272 to 3,291 and (nt) -8 to 12/3,796 to 3,815 (relative to the start codon), respectively (Supplementary Table S1). The resulting 3.3- kb and 3.8-kb cDNA amplicons were cloned and sequenced.

### Phylogenetic Analysis of the *C. parasitica* C5-DNA Methyltransferases

The phylogenetic relationships of *CpDmt1* and *CpDmt2* with representative fungal cytosine-specific methyltransferase domains were analyzed using MEGA7 (Kumar et al., 2015). The maximum likelihood method based on the JTT matrix-based model was used to estimate the evolutionary history (Jones et al., 1992).

The protein domain features were predicted and compared among the selected fungal methyltransferases using batch CD-search of the NCBI Conserved Domain Database CDD (Marchler-Bauer and Bryant, 2004; Marchler-Bauer et al., 2015).

### Quantitative Analysis of Transcript Accumulation Using Real-Time RT-PCR

To examine the expression levels of target genes, quantitative RT-PCR was performed (Park et al., 2012). Briefly, a GeneAmp 7500 sequence detection system (Applied Biosystems, Foster City, CA, United States) using a SYBR green mixture RT kit (Applied Biosystems) was applied. 1 μg of total RNA was treated with RNase-free RQ1 DNaseI and then was reverse transcribed. Real-time PCR was performed on 2 μL of reverse-transcribed cDNA with 1 μL of each forward and reverse primer at 150 nM concentration. The glyceraldehyde-3-phosphate dehydrogenase gene (*Gpd*) was used as an internal control. Analyses were conducted at least two independent RNA preparations, in triplicate for each transcript, with primers specific for *Gpd* and the target genes. Primer pairs for each gene are shown in Supplementary Table S1. Transcript abundance, relative to the amount of *Gpd*, in the sample was calculated based on the fold change expression of target genes normalized to the internal control *Gpd* (Parsley et al., 2002). RNA was extracted from liquid cultures as described previously (Kim et al., 1995).

### Disruption of the *C. parasitica CpDmt1* and *CpDmt2* Genes

The replacement vectors pDDMT1 and pDDMT2, which were designed to favor double-crossover integration events, were constructed. In the replacement vector pDDMT1, the hygromycin phosphotransferase gene cassette (*hph*) was inserted between sites nt 15 and 1,968 of the *CpDmt1* gene relative to the start codon and flanked by approximately 1.7 and 2.0 kb of 5′ and 3′ sequences, respectively. The 5.6-kb *CpDmt1* disruption cassette was then used to transform the virus-free EP155/2 strain. In a similar way, in the replacement vector pDDMT2, the hygromycin phosphotransferase gene cassette (*hph*) was inserted between sites nt 1 and 3,367 of the *CpDmt2* gene relative to the start codon and flanked by approximately 2.0 and 1.9 kb of 5′ and 3′ sequences, respectively. The 7.3-kb *CpDmt2* disruption cassette was then used to transform the virus-free EP155/2 strain.

Functional complementation of the *CpDmt1*-null mutant using a wild-type allele was performed. The complementing vector pCDMT1 was constructed by insertion of *Spe*I/*Sac*II-digested pCDMT1 carrying a 5.6-kb fragment containing the full-length *CpDmt1* gene into *Spe*I/*Sac*II digested pBSSKG plasmid containing the geneticin resistance cassette of pSilent-Dual1G (pSD1G) in pBluescriptII SK(+) (Nguyen et al., 2008). The resulting vector was then used to transform the *CpDmt1*-null mutant. Functional complementation of the *CpDmt2*-null mutant was carried out in a similar fashion. The complementing vector, pCDMT2, was constructed by inserting *Hin*dIII/*Spe*I-digested pCDMT2 carrying a 7.0-kb fragment containing the full-length *CpDmt2* gene into the pBSSKG plasmid digested with *Hin*dIII and *Spe*I, followed by transformation into the *CpDmt2*-null mutant.

### Southern Blot Analysis

Genomic DNA was isolated from *C. parasitica* as described previously (Churchill et al., 1990). DNA (10 μg) was digested with the appropriate restriction enzyme, blotted onto a nylon membrane, and hybridized with radioactive-labeled probes (Sambrook et al., 1989).

### Characteristics of the *CpDmt1* and *CpDmt2*-Null Mutants

The phenotypic and molecular characteristics of the *CpDmt1-* and *CpDmt2*-null mutants were examined with the comparison of wild-type EP155/2 and the hypovirulent UEP1 strains. Phenotypic changes, such as growth rate, pigmentation, conidiation, and mating capability, were measured as described previously (Powell and Van Alfen, 1987; Kim et al., 2002). Morphological characteristics were examined on PDAmb medium.

### Statistical Analysis

All statistical analyses were performed in SPSS version 22.0 (SPSS Inc., Chicago, IL, United States). Pearson’s Chi-square test was used to evaluate the difference in methylation frequency between strains. One-way ANOVA was used to analyze the significant differences of relative gene expression.

## Results

Although the CWI signal transduction pathway was shown to play a role in sectorization, the molecular basis of sectorization in the *CpBck1*-null mutant remained unclear (Kim et al., 2016). As only a restricted region of the mycelia on plates played a role in sectorization, stable inheritance of the sectored phenotype, once sectored, was maintained, and genome integrity was not disturbed by the *Crypt1* transposable element (GenBank no. AF283502), epigenetic changes in sectored progeny of the *CpBck1*-null mutant, especially in relation to DNA methylation, were examined by the WGBS (Kim et al., 2016).

### DNA Methylation Levels

Quantification of global DNA methylation was performed using a MethylFlash^TM^ Methylation DNA Quantification Kit (Epigentek). Total DNA from the wild-type EP155/2, *CpBck1*-null mutant (TdBCK1), sectored progeny of the *CpBck1*-null mutant (TdBCK1-S1), and the complemented strain of the sectored *CpBck1*-null mutant strains (TcBCK1-S1) were prepared (Kim et al., 2016). As shown in Supplementary Figure S1, DNA methylation was detected in all four strains. Interestingly, there were differences in global DNA methylation among strains; the optical density of methylated DNA ranged from 0.150 to 0.231 among samples, in the order of wild-type EP155/2, TdBCK1, TcBCK1-S1, and TdBCK1-S1. Interestingly, the methylation levels of the mutant strains TdBCK1, TcBCK1-S1, and TdBCK1-S1 were significantly lower than that of the wild-type (*p* < 0.05, one-way ANOVA).

### Whole-Genome Bisulfite Sequencing of Four Strains

Whole-genome bisulfite sequencing enables analysis of the methylation state of all cytosine residues in an individual DNA sequence (Clark et al., 2006; Xiang et al., 2010; Jeon et al., 2015). Thus, WGBS of genomic DNAs extracted from mycelia of the wild-type, TdBCK1, TdBCK1-S1, and TcBCK1-S1 strains was performed by next-generation sequencing (NGS) using an Illumina HiSeq 2000 system. Read pairs were subsequently mapped to the corresponding reference genome using BisMark (Nuss et al., 2010; Krueger and Andrews, 2011). These procedures resulted in 49–57 million reads corresponding to 9.8, 10.6, 11.5, and 10.6 Gb high-quality sequences, respectively, with average map rates of 67–81% in read alignment (Supplementary Table S2). This represented approximately 75× to 99× coverage of the *C. parasitica* genome, the reference sequence of which has an estimated size of 43.9 Mb. Lambda DNA, which lacks DNA methylation, was included in the bisulfite treatment to estimate the conversion rate (Remnant et al., 2016). In our experiment, the conversion rate was at least 99.4%. Additionally, to minimize the bias that may result from the overrepresentation per site, sites read more than 500 times or fewer than five times were excluded from the analysis, which allowed the methylation levels of individual sites to be determined with reasonable confidence. The average map rates of cytosine sites analyzed after the coverage cutoff (5–500) were 71.9, 66.7, 80.1, and 75.5% in read alignment for the wild-type, TdBCK1, TdBCK1-S1, and TcBCK1-S1 strains, respectively.

Whole-genome bisulfite sequencing data were validated by cloning and sequencing PCR products from bisulfite-treated DNA and analysis by CyMATE for two representative [scaffold_13: 32825-32935 and scaffold_13: 33015-33109] loci predicted to carry methylation (Supplementary Table S3). Comparison of two independent datasets showed that over 90% of the methylated cytosine (mC) sites identified in our WGBS overlapped with mCs on CyMATE analysis, suggesting the sensitivity and reliability of the WGBS performed here.

### Genome-Wide DNA Methylation Patterns of Four Strains

The global methylation level was estimated by dividing the amount of methylated cytosine by the total amount of cytosine detected in each sample (mC/total C). The global methylation level of the wild-type was 3.9%, which was low but sufficiently significant (*p* < 0.001, Chi-square test) and within the range in other fungal species (2–6%). However, changes in the global methylation levels were observed in mutant strains. The global methylation level of TdBCK1-S1 was lowest, and only 1.0% of detected cytosines were methylated. Interestingly, the complemented strain TcBCK1-S1 showed restoration to a level of 2.1%. The parental mutant strain TdBCK1 showed an intermediate level of 2.6%. However, global DNA methylation levels are only an indirect indicator of the mCs in the genome. Therefore, further analyses were conducted to determine the variation in number of mC sites among the four strains (Supplementary Table S4).

In total, 1,410,293 cytosine sites were identified as mC sites in the wild-type EP155/2, accounting for approximately 6.4% of all genomic cytosine sites that experienced methylation. However, more mC sites were observed in the mutant strains. The parental TdBCK1 strain showed the most mC sites, with a total of 1,694,399 sites, accounting for approximately 7.7% of all genomic cytosines. Considering the least average map rate of 66.7% (Supplementary Table S2) and the most mC sites among the four strains, the increased number of mC sites in the TdBCK1 strain was dramatic. The complemented TcBCK1-S1 and sectored TdBCK1-S1 strains showed an intermediate number of mC sites (1,576,353 and 1,575,942, respectively). Considering the average map rates of 75.5 and 80.1% covered in the TcBCK1-S1 and TdBCK1-S1 strains, respectively, which were higher than 71.9% in the wild-type strain, the increased number of mC sites may not be as dramatic as that of TdBCK1. However, these two mutant strains still showed an increased number and proportion of mC sites compared to wild-type. Thus, it is interesting that the wild-type EP155/2, which contains the highest global methylation level among the four strains, showed the fewest number of mC sites. In addition, the sectored progeny (TdBCK-S1) exhibited marked genome-wide DNA hypomethylation but an increase in methylation sites. *C. parasitica* showed cytosine methylation in any DNA context, including symmetrical CG and CHG (where H = A, T, or C) and asymmetrical CHH, as in other species of ascomycetes (Zemach et al., 2010; Aramayo and Selker, 2013). The methylation proportions representing the ratio among all three classes of potential methylation site in the wild-type were 71.6, 21.8, and 6.6% for CHH, CHG, and CG, respectively (Supplementary Table S4). These proportions were quite different from those expected based on random frequencies of 56, 19, and 25%, respectively, and indicated strong methylation preference for CHH and CHG sites. However, methylation proportions of all three classes of potential methylation site were similar in all four strains. They were, in order, CHH (68.7–71.6%), CHG (21.8–22.6%), and CG (6.6–8.9%); therefore, they represented epigenetic homogeneity with respect to all three classes of potential methylation site. The global methylation level of CHH and CHG sites in the wild-type strain was 4.3%, while those of CHG and CHH sites were 2.7 and 3.0% in the parental TdBCK1, 1.1 and 1.1% in TdBCK1-S1, and 2.3 and 2.4% in TcBCK1-S1, respectively. The global methylation levels of CG sites were 0.09, 0.07, 0.05, and 0.06% for the wild-type EP155/2, TdBCK1, TdBCK1-S1, and TcBCK1-S1 strains, respectively, which were also markedly lower than their corresponding global methylation levels.

**FIGURE 1:**
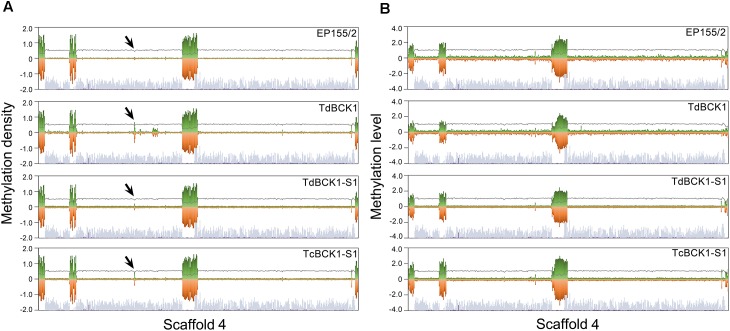
Representative chromosomal distribution of DNA methylation in the scaffold 4. The density **(A)** and level **(B)** of DNA methylation are depicted by green and red bars indicating methylation in Watson and Crick strands, respectively. The steel blue and purple bars indicate the genic region and TEs at the corresponding chromosome, respectively. The gray lines indicate CG contents. Arrows indicate the regions showing a difference among strains. Note that the densely methylated regions are located at or around gene-poor region of corresponding chromosome.

As shown in Supplementary Figure S2, mC sites in the genome of the wild-type were not evenly distributed, but rather formed relatively densely methylated domains. Moreover, when compared with the gene maps showing the density of putative genes, these densely methylated regions were located mostly around intergenic regions and gene-poor regions across contigs (**Figure 1**). Interestingly, the clustering pattern of mC sites was similar among all four strains except for the domain in supercontig 4, which showed the appearance of a new densely methylated domain in the mutant strains compared to the wild-type strain (Supplementary Figure S2). Considering the differences in global methylation level and the number of mC sites in the wild-type strain, the differences in DNA methylation patterns among the strains were ascribed to changes in both the methylation level (defined as the number of mC reads divided by the number of total reads covering the site) of each individual mC site and the density (defined as the number of mC sites per 10 kb) of mC sites. However, similarities in the patterns of mC site clusters among the strains suggest that changes in mC sites are restricted to areas around the mC site clusters. The differentially methylated domain in supercontig 4 of the mutant strains is interesting because it is not located in the gene poor region and was identified as a new cluster. Thus, we examined the differentially methylated genes in this specific domain (supercontig 4: 1,530,000–1,540,000) and found that four predicted genes (Supplementary Table S5). Among these four predicted genes, one was suggested to be a transmembrane transporter and three are without any known protein domain. Although these results prompt further investigation, we hypothesize there may be significant changes in the metabolites of the mutant strains from those of the wild-type strain. Interestingly, adjacent to the mutant-specific differentially methylated region, there are highly methylated regions specific for the TdBCK1 strain. Sequence analysis of the three most highly methylated regions specific for TdBCK1 (supercontig 4: 1,840,000–1,850,000, 1,870,000–1,880,000, and 1,920,000–1,930,000) revealed six predicted genes. Among these, one was identified as a gene containing a domain involved in carbohydrate metabolic process while the others did not have any specific domain (data not shown).

**FIGURE 2:**
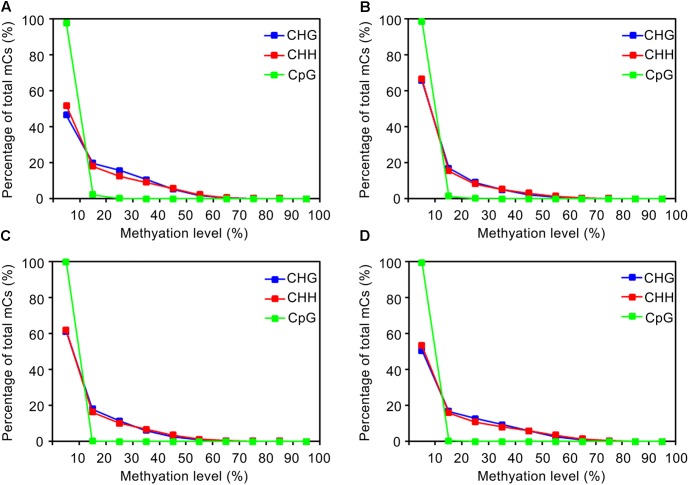
Distribution of DNA methylation levels depending on the sequence context in the genome. **(A–D)** Represent the wild-type EP155/2, TdBCK1, TdBCK1-S1, and TcBCK1-S1 strains, respectively.

The distribution of methylation levels for each mC site was analyzed. As shown in **Figure 2**, the methylation levels of individual mC sites remained less than 70% across samples, with a strong preference for ≤40%. Although the proportions of mC sites with different methylation levels were similar across samples, i.e., a sharp decrease from the ≤10% level to the next level of 20% and then a gradual decrease to 70%, the wild-type strain showed a larger proportion of the ≥20% methylation level (**Figure 2A**) compared to the other three strains (**Figures 2B–D**). These results were in agreement with the results of the global methylation levels. Moreover, the methylation levels of CG sites were mostly ≤10% among all strains. Similar results were found in *Uncinocarpus reesii* and *N. crassa* (Zemach et al., 2010; Aramayo and Selker, 2013). Thus, methylation occurred less frequently in the CG sites and the methylation level at individual CG sites, if any, showed a strong preference for ≤10% (Supplementary Table S4), which resulted in markedly lower global level of methylation at the CG sites than at the CHH and CHG sites. Additionally, considering the fewer number of mC sites, low methylation levels, and relatively small changes in the methylation levels in the CG sites among strains, hypomethylation of mC sites in mutant strains occurred mainly in the CHH and CHG rather than the CG context.

**FIGURE 3:**
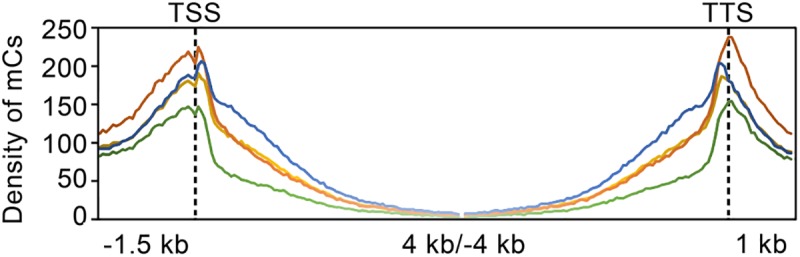
Density of DNA methylation sites in and around genes in the genome. Green, orange, blue, and yellow lines indicate the wild-type, TdBCK1, TdBCK1-S1, and TcBCK1-S1, respectively.

The distribution of mC sites to different genomic features such as intergenic regions, genes (exons and introns), and upstream (1.5 kb, relative to the putative start codon) and downstream (1.0 kb, relative to the putative stop codon) of genes were analyzed by calculating and comparing DNA methylation densities of the whole genome (Supplementary Table S6). Compared to the intergenic region encompassing 70% of methylation sites, the proportion of mC sites in genic regions in the wild-type was only 30%, which was significantly low. Interestingly, TdBCK1 and its sectored progeny, TdBCK1-S1, showed the decreased proportions of mC in intergenic regions of 62 and 61%, respectively. The complemented strain, TcBCK1-S1, showed an intermediate proportion (65%) of mC sites in intergenic regions. Within genic regions of the wild-type strain, methylation was found in both coding and non-coding regions including upstream and downstream regions (**Figure 2**). Interestingly, methylation peaked in the upstream and downstream regions of genes and a sharp drop was observed at the boundaries of coding regions of all strains. Methylation within coding regions (gene body methylation) was observed mostly near the start and end of coding regions, gradually disappeared, and depleted at the center in all strains (**Figure 3**). The distribution of mC sites in different genomic features of other strains was similar to that of the wild-type. Compared to the wild-type, these increased proportions of mC sites were mainly found in exon regions. For example, compared to the wild-type showing 7.3% exon methylation sites, TdBCK1-S1 showed a marked increase to 15.1% and almost all the increased proportion of mC sites was found in near the start and end, but not the middle, of coding regions. Interestingly, when we measured the methylation levels around genic regions depending on the DNA context, hypomethylation was observed in the mutant strains compared to the wild-type strain. In addition, the methylation levels in exon regions of all strains were lower than any other genic features, i.e., upstream, downstream, and intron. Thus, although marked increases in the number of mC sites were mostly observed in the exon regions of the mutant strains, the methylation levels around genes of the mutant strains did not change significantly, i.e., still lower than those of the wild-type strain and the methylation levels in exon regions were lower than other genic regions.

**FIGURE 4:**
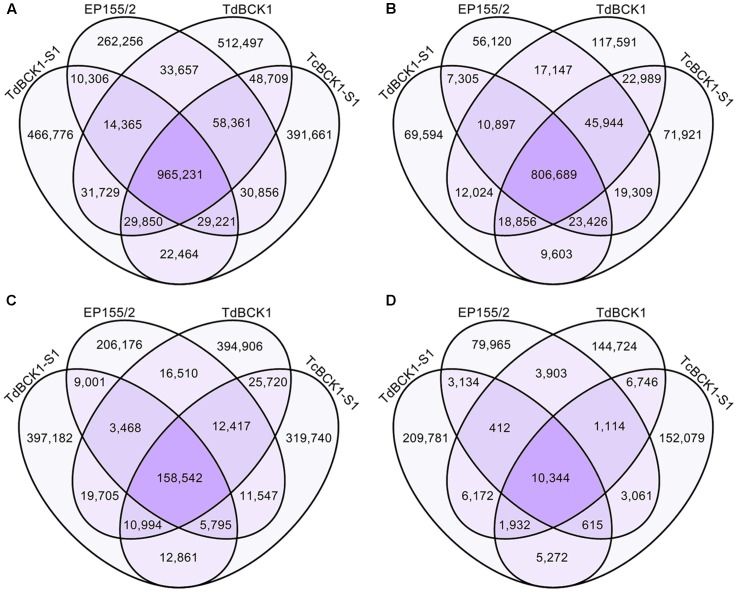
Venn diagram of DNA methylation sites depending on genic features in the genome. **(A–D)** Represent the distribution of mC sites in the whole genome, intergenic regions, genic regions, and exons within genic regions, respectively.

Among all mC sites, 965,231 sites were common to all four strains (**Figure 4A**). Additionally, there were large numbers of mC sites that were common to two or three strain combinations. Furthermore, a number of strain-specific mC sites were identified; that is, 262,256, 512,497, 466,776, and 391,661 mC sites were specific for the wild-type, TdBCK1, TdBCK1-S1, and TcBCK1-S1, respectively, which represented 19, 30, 30, and 25% of the total, respectively. Among the common 965,231 mC sites, 84% were found in intergenic regions (**Figure 4B**). However, most of the strain-specific mC sites were found in genic regions; that is, 79, 77, 85, and 82% of the mC site specific for the wild-type, TdBCK1, TdBCK1-S1, and TcBCK1-S1, respectively, were in genic regions (**Figure 4C**). Within the genic regions, exons had the highest proportion (28–45%) of strain-specific mC sites (**Figure 4D**). It will be of interest to see how these strain-specific mC sites contribute to gene expression resulting in phenotypic changes.

In summary, compared with those of the wild-type strain, decreased global methylation levels and an increased number of mC sites were observed in the mutant strains. Methylation occurred in any DNA context, such as CG, CHG, and CHH, at a similar proportion in all four strains. The distribution of mC sites was not even, but clustered around intergenic and gene-poor regions across the contigs, with a notable exception of a domain in supercontig 4 of the TdBCK1 strain. In terms of genomic features, the distribution of mC sites demonstrated a preference for intergenic to genic regions. However, an increased distribution of mC sites in the genic regions was observed in the mutant strains, and these increased mC sites were mainly confined in exon regions. There were common mC sites among all four strains, but also strain-specific mC sites, which were mostly found in intergenic and genic regions, respectively. The methylation level of each mC site was not high, but showed a strong preference for ≤40% in all strains.

### Transcriptional Profiling of Two Putative DNMTase-Encoding Genes

Quantification of the global DNA methylation level indicated the considerable changes in the DNA methylation of TdBCK1-S1. Therefore, we reasoned that the molecular mechanism of sectorization involves altered DNA methylation and investigated the expression profiles of the gene encoding DNA methyltransferase (DNMTase). A search for the DNMTase gene in the annotated *C. parasitica* draft genome sequence (Nuss et al., 2010) yielded three C5-DNA-methyltransferase genes, among which, one in scaffold_3:721439-722781 of the EP155 genome sequence assembly had a truncated C5-DNA-methylase domain. In addition, no corresponding RNA transcript was identified. Thus, considering it as a pseudogene, the other two genes designated as *CpDmt1* (scaffold_2:1971293-1972939) and *CpDmt2* (scaffold_6:348508-351624) were further analyzed for their functional contribution toward the DNA methylation mediated sectorization process. Based on the results of genomic sequence analysis, near full-length cDNA clones for *CpDmt1* and *CpDmt2* were obtained using RT-PCR with the primer pairs CpDmt1-mF1/CpDmt1-mR1 and CpDmt2-mF1/CpDmt2-mR1, respectively. Sequence comparison with the corresponding genomic sequences revealed that both *CpDmt1* and *CpDmt2* genes were intronless. The deduced *CpDmt1* protein product (CpDMT1) consisted of 1,095 amino acids, with an estimated molecular mass of 121.1 kDa and pI of 5.63 (GenBank No. MF000328). The *CpDmt2* gene consisted of two exons, with one intervening sequence of 30 bp. The deduced *CpDmt2* protein product (CpDMT2) consisted of 1,259 amino acids, with an estimated molecular mass of 141.8 kDa and pI of 5.56 (GenBank no. MF000329). Phylogenetic analysis indicated that the cloned *CpDmt1* and *CpDmt2* genes, showing high similarity to repeat-induced point mutation (RIP) defective (RID) and defective in DNA methylation (DIM-2) of *N. crassa*, respectively, belonged to DNMT subfamilies of dnmt4 and dnmt1, respectively (Supplementary Figure S3 and Supplementary Table S7; Freitag et al., 2002; Ponger and Li, 2005).

**FIGURE 5:**
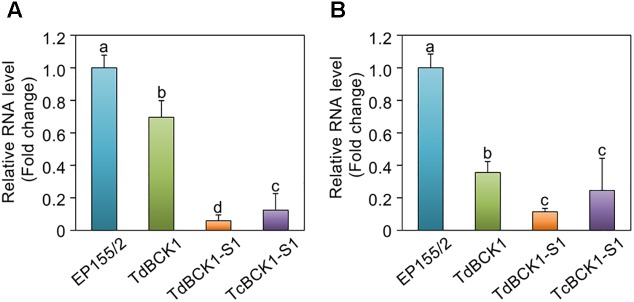
Analysis of *CpDmt1* and *CpDmt2* expression in the wild-type EP155/2, TdBCK1, TdBCK1-S1, and TcBCK1-S1 strains. **(A)** Quantitative real-time RT-PCR analysis of *CpDmt1* transcript levels relative to those of the glyceraldehyde-3-phosphate dehydrogenase (*Gpd*) gene. The values on the *y*-axis were represented with standard deviations, based on three independent measurements of two independent RNA preparations, indicated by the error bars. Different letters indicate significant differences between strains according to ANOVA at *p* = 0.01. **(B)** Quantitative real-time RT-PCR analysis of *CpDmt2* transcript levels. Same procedure and statistical analysis as **(A)** were applied.

Quantitative real-time RT-PCR was performed to obtain the expression profiles of *CpDmt1* and *CpDmt2*. As shown in **Figure 5**, *CpDmt1* (**Figure 5A**) and *CpDmt2* (**Figure 5B**) expression levels were markedly down-regulated in the sectored progeny TdBCK1-S1. Additionally, the parental TdBCK1 and complemented TcBCK1-S1 strains showed significantly reduced (*p* < 0.01, one-way ANOVA) expression of both *CpDmt1* and *CpDmt2* compared to the wild-type. These results indicated that hypomethylation of mutant strains was correlated with the expression levels of both *CpDmt1* and *CpDmt2*.

### Phenotypic Changes in the Two DNMTase Mutant Strain

As the expression levels of both *CpDmt1* and *CpDmt2* were affected in TdBCK1-S1, we constructed *CpDmt1*- and *CpDmt2*-null mutants to examine the phenocopy of the sectorization. Eighty and 90 transformants for each *CpDmt1*- and *CpDmt2*-null mutant, respectively, were screened by PCR using a pair of outer gene-specific and inner *hph* primers (DMT1_EXT_F1&DMT1_EXT_R2 and DMT2_EXT_F1& DMT2_EXT_R2 in Supplementary Table S11) corresponding to -1,765 to -1,747 and 4,397 to 4,415 (relative to the start codon of *CpDmt1*) and -2,464 to -2,482 and 5,577 to 5,595 (relative to the start codon of *CpDmt2*), respectively. Transformants showing PCR amplicons of the expected size of 6.8 and 7.3 kb corresponding to the *CpDmt1*- and *CpDmt2*-null mutants, respectively, were obtained. These putative *CpDmt1*- and *CpDmt2*-null mutants were single-spored and single-spored progenies were further confirmed by Southern blotting analysis (Supplementary Figure S4).

**FIGURE 6:**
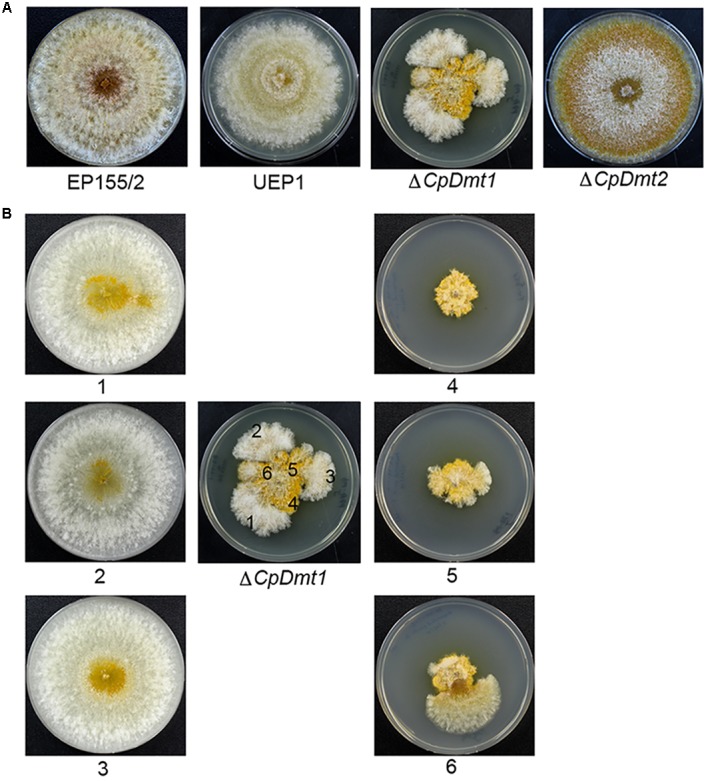
Colony morphology of the *CpDmt1-* and *CpDmt2*-null mutant strains. **(A)** Colony morphology of the mutant strains with comparison to the wild-type EP155/2 and CHV1-infected hypovirulent UEP1 strains. The *CpDmt1*-null mutant showed sporadic appearance of sectorization at the colony margin after 10 days of culture, while the *CpDmt2*-null mutant showed a near absence of aerial mycelia around the margins of colony. **(B)** Colony morphology of the progenies of the *CpDmt1*-null mutant. Numbers indicate colonies of the corresponding regions after 7 days of successive culture. The sectored phenotype was maintained in successive cultures and the non-sectored area showed the characteristic parental-mutant phenotype.

The *CpDmt1*-null mutant showed retarded growth (**Figure 6A**) compared to the wild-type. However, compared to the *CpBck1*-null mutant showing severely retarded growth with thinner invasive feeding hyphae, the near-absence of the typical mycelial mat and aerial hyphae on the surface, and the absence of typical spore-forming structures, the *CpDmt1*-null mutant showed abundant sporulation, active mycelial growth and near-normal pigmentation (Kim et al., 2016). When the *CpDmt1*-mutant culture was aged, it started to show sectorization with robust mycelial growth (**Figure 6B**). The sectored progeny maintained the characteristics of active mycelial growth with reduced sporulation and restricted pigmentation. The sectored progeny differed from the wild-type in that fluffy mycelial growth was maintained and differentiation, such as pigmentation and sporulation, occurred but to a lesser extent than in the wild-type. In contrast, minor changes were observed in the *CpDmt2*-null mutant (**Figure 6A**). The *CpDmt2*-null mutant showed a similar growth rate to the wild-type. However, aerial hyphae around the colony margin disappeared with concurrent uncovering of pigmented spore-bearing structures, which differed from the wild-type showing the usual disappearance of aerial mycelia from the center (**Figure 6A**).

We, next examined DNA methylation of the *CpDmt1*- and *CpDmt2*-null mutants by measuring the changes in DNA methylation of representative genes using CyMATE. CyMATE indicated that both mutants showed reduced levels or near absence of DNA methylation (Supplementary Table S3). These results indicated that both genes are involved in DNA methylation. Two DNMTases, MoDIM-2 and MoRID, which are homologs of DIM-2 and RID, respectively, were observed in *Magnaporthe oryzae* (Jeon et al., 2015). Unlike the RID in *N. crassa* showing no evidence of DNMTase activity, both genes were suggested to be involved in DNA methylation, either alone or in combination. Moreover, it was interesting that the colony morphology of the MoDIM-2-null mutant in *M. oryzae* was fluffier than that of the wild-type. These results suggested that robust growth of mycelia, one of the signature characteristics of sectorization, is a common phenomenon governed by a set of genes controlled by changes in DNA methylation. However, changes in DNA methylation were specific depending on the DNMTases and resulting in different phenotypes as evidenced by the differences between *CpDmt1*- and *CpDmt2*-null mutants and other fungi.

## Discussion

Epigenetic modifications serve as a connection between genetic components and environmental changes (Flanagan et al., 2006; Bock and Lengauer, 2008). Although not all fungi have a significant level of DNA methylation in their genomes (Liu et al., 2012), genome-wide DNA methylation has been profiled in several fungi using bisulfite sequencing (Mishra et al., 2011; Jeon et al., 2015). The present study contributed to our understanding of the DNA methylation status of *C. parasitica*. The global methylation level of the wild-type was 3.9%, which was low but sufficiently significant. In addition, the methylation context was not limited to the symmetrical CG or CHG, but also included CHH. In contrast, the frequency of 5-mC at CG dinucleotide sites was rare, accounting for only 1.0% of the total CG sites, which strongly suggested the existence of an active C/G to T/A conversion mechanism. The methylation level of each mC site was not high, mostly ranging from 10 to 40%. Moreover, the methylation levels of CG sites were mostly ≤10% among all strains. These results explain why we were not able to identify differences in DNA methylation patterns using methylation-sensitive isoschizomers, such as *Msp*I/*Hpa*II, which are sensitive to the C residue of CG dinucleotides in the recognition sequence CCGG. Although mC sites were more common in the intergenic region, gene body methylation was also observed (Zemach et al., 2010; Jeon et al., 2015).

Interestingly, our CWI mutant showed sporadic sectorization as the culture aged, and the sectored progeny maintained the characteristics of sector (Kim et al., 2016). Our WGBS indicated marked changes (a nearly fourfold difference, i.e., 3.9% vs. 1.0%) in global methylation level in the sectored progeny of the *CpBck1* mutant. These results indicated that fungal sectorization was associated with epigenetic changes, and global hypomethylation was observed. Considering changes in mC site, the mutant strains showing a decreased global methylation level had an increased number of mC sites compared to the wild-type. Additionally, although there were 19–30% strain-specific mC sites, most mC sites overlapped with those in the wild-type. Accordingly, most 5-mC sites were hypomethylated in the mutant strains, and the sectored progeny showing lowest global methylation level of 1.0 and 30% of specific sites appeared to be the most hypomethylated strain. These changes in the DNA methylation profile among strains suggested that DNA methylation is involved in the changes of gene expression resulting in different phenotypes.

To investigate the possible molecular mechanism involved in the epigenetic changes in the mutants of the CWI MAPK signaling pathway, such as TdBCK1 and its sectored progeny, we examined the expression characteristics of genes responsible for DNA methylation and phenocopied the mutant characteristics, such as sectorization, by mutating the corresponding genes. We identified two DNMTases, *CpDmt1* and *CpDmt2*, representing RID/Masc1-like and DIM-2-like, respectively. The specificity of the existing DNMTase suggests that the CG context is not the preferential target, which explains the low level and density of methylation in the CG context (Amselem et al., 2015). In addition, transcription analysis indicated that transcripts of both genes were down-regulated in the sectored progeny with hypomethylation. Although there are genes involved in active DNA demethylation, such as those genes encoding the putative ten–eleven translocation (TET) protein (Kohli and Zhang, 2013; Zhang et al., 2014) and DNA glycosylase (Zhu, 2009) in the genome of *C. parasitica*, transcriptional analysis of these two DNMTases suggested that hypomethylation of the mutant strains is due to passive demethylation by the down-regulation of these DNMTases. Furthermore, the *CpDmt1* mutant, although not as distinctive as that of the sectored progeny of the *CpBck1*-null mutant, phenocopied sectorization at the margins of the colonies, and the progeny from the sectored area maintained the characteristics of the sector, including robust mycelial growth, reduced conidiation, and restricted pigmentation. In addition, CyMATE analysis indicated that the *CpDmt1*-null mutant showed a reduced level of methylation. These results clearly indicate that changes in DNA methylation result in sectorization, and we demonstrate the genetic background of sectoring, i.e., *CpDmt1* plays an important role in DNA methylation accompanied with sporadic sectorization. It is interesting that, even though both genes were down-regulated in the sectored progeny of the *CpBck1*-null mutant, only the *CpDmt1*-null mutant, but not the *CpDmt2*-null mutant, showed sectorization, and the *CpDmt2*-null mutant showed different phenotypeswith reduced methylation levels. These results strongly suggest functional specificity depending on the DNMTase, and it will be interesting to analyze genes affected by the mutation of each DNMTase gene. Moreover, further methylome analyses will aid in our understanding of the epigenetic changes responsible for the specific phenotypic changes.

## Conclusion

The present study revealed the characteristics of genome-wide DNA methylation in chestnut blight fungus *C. parasitica*. Additionally, we showed that sectorization due to mutation in the *CpBck1* gene was accompanied by changes in DNA methylation, with decreased global methylation level but increased mC sites. We showed that the expression of DNMTase genes was severely affected in the sectored progeny of the *CpBck1-*null mutant. Mutants in one of two DNMTase genes also showed sectorization. These results clearly indicated that the CWI signal transduction pathway is important for maintaining DNA methylation via DNMTases and changes in DNA methylation resulted in specific phenotypic changes of sectorization. Our research will expand understanding of the epigenetic changes due to the well-known signal transduction pathway and clarify the molecular mechanism of fungal debilitation such as sectorization.

## Availability of Supporting Data

The raw sequencing data of WGBS are deposited in SRA at NCBI (https://submit.ncbi.nlm.nih.gov/subs/sra/) with the accession number SRP103703.

## Author Contributions

D-HK supervised the experiments. JeC, and J-MK designed the experiments. JHH, JJ, JaC, and Y-HL designed WGBS and analyzed the data. K-KS, JB, and Y-HK performed gene replacement experiments and functional analysis of mutants. D-HK, J-MK, and K-KS prepared the figures and edited the manuscript. D-HK wrote the manuscript.

## Conflict of Interest Statement

The authors declare that the research was conducted in the absence of any commercial or financial relationships that could be construed as a potential conflict of interest.

## Footnotes

1 http://genome.jgi-psf.org/Crypa2/Crypa2.home.html
