# Supplementary material for: Global DNA Methylation in the Chestnut Blight Fungus Cryphonectria parasitica and Genome-Wide Changes in DNA Methylation Accompanied with Sectorization
Source: Front Plant Sci. 2018 Feb 2;9:103. doi: 10.3389/fpls.2018.00103 (PMC5801561; doi:10.3389/fpls.2018.00103)
Supplement: Supplementary file 1 [file Table_1.DOCX]

**Supplemental Table S1.** List of PCR primer sequences

| Primer | Sequence (5’ to 3’) | Use |
| --- | --- | --- |
| CPDMTC5_2549_F1 | TCCTTCATCATCACAACAAGCA | Construction of *CpDmt1*-null mutant |
| CPDMTC5_2549_R1 | CGAGCTTGGCGTAATCATGGTTGGCAGCGGTGGTGGCAGC | Construction of *CpDmt1*-null mutant |
| CPDMTC5_2549_F2 | TTCACTGGCCGTCGTTTTACAAGTCGTTCAGACATATTGG | Construction of *CpDmt1*-null mutant |
| CPDMTC5_2549_R2 | AACAAGTATGGTGTCTACATCGA | Construction of *CpDmt1*-null mutant |
| CPDMTC5_1891_F1 | GGACAGAAAAGCAGTGACGG | Construction of *CpDmt2*-null mutant |
| CPDMTC5_1891_R1 | ATGGTCATAGCTGTTTCCTGAAGATACCCGATTGCTTCTG | Construction of *CpDmt2*-null mutant |
| CPDMTC5_1891_F2 | AATTCACTGGCCGTCGTTTTACAATAGCGCAAAGTCTGAG | Construction of *CpDmt2*-null mutant |
| CPDMTC5_1891_R2 | TCACAGTGCAGCCGATCTC | Construction of *CpDmt2*-null mutant |
| HYG_F1_2549 | CCAATATGTCTGAACGACTTGTAAAACGACGGCCAGTGAA | Construction of *CpDmt2*-null mutant |
| HYG_R2_2549 | GCTGCCACCACCGCTGCCAACCATGATTACGCCAAGCTCG | Construction of *CpDmt2*-null mutant |
| HYG_F1_1891 | CTCAGACTTTGCGCTATTGTAAAACGACGGCCAGTGAATT | Construction of *CpDmt2*-null mutant |
| HYG_R2_1891 | CAGAAGCAATCGGGTATCTTCAGGAAACAGCTATGACCAT | Construction of *CpDmt2*-null mutant |
| DMT1_EXT_F1 | GGACGAGGACCTGCCCTA | Cloning of *CpDmt1* gene/  Screening of *CpDmt1*-null mutant |
| DMT1_EXT_R2 | CTACTCGTGTGGCTTCTA | Cloning of *CpDmt1* gene/  Screening of *CpDmt1*-null mutant |
| DMT2_EXT_F1 | GACGACCTCGTGGACAAG | Cloning of *CpDmt2* gene/  Screening of *CpDmt2*-null mutant |
| DMT2_EXT_R2 | AGCGTCACCCGAGATCTC | Cloning of *CpDmt2* gene/  Screening of *CpDmt2*-null mutant |
| CpDmt1-mF1 | GATCCAATATGTCTGAACGACT | cDNA cloning of *CpDmt1* |
| CpDmt1-mR1 | TCGCACTTACAAGTTTTCCT | cDNA cloning of *CpDmt1* |
| CpDmt2-mF1 | GCGCTATTATGGAGCCATTA | cDNA cloning of *CpDmt2* |
| CpDmt2-mR1 | GAAGGATATCAAAGTAAGCA | cDNA cloning of *CpDmt2* |
| Gpd_RT_F1 | CCGTCAACGACCCCTTCAT | qRT-PCR |
| Gpd_RT_R1 | GTTGCCGTGTTGAGAGTCATACTT | qRT-PCR |
| CpDmt1_RT_F1 | ATGGCTATAAGTTGGTAG | qRT-PCR |
| CpDmt1_RT_R1 | CCTATTTCTAATACAGTCG | qRT-PCR |
| CpDmt2_RT_F1 | GCTATTCACACCTATATG | qRT-PCR |
| CpDmt2_RT_R1 | CTAAACTTACCTTCTATCC | qRT-PCR |
| Bisulfite_F1 | TTTTTGAAAGTAATGAATA | BS-PCR^a^ |
| Bisulfite_R1 | CATTTATAAATTCCTTCCAAC | BS-PCR |

^a^Sequencing of PCR product using bisulphite-treated gDNA for CyMATE analysis.
