# Supplementary material for: Global DNA Methylation in the Chestnut Blight Fungus Cryphonectria parasitica and Genome-Wide Changes in DNA Methylation Accompanied with Sectorization
Source: Front Plant Sci. 2018 Feb 2;9:103. doi: 10.3389/fpls.2018.00103 (PMC5801561; doi:10.3389/fpls.2018.00103)
Supplement: Supplementary file 2 [file Table_2.DOCX]

**Supplemental Table S2.** Read alignment of whole genome bisulfite sequence (WGBS)

| Strain | EP155/2 | TdBCK1 | TdBCK1-S1 | TcBCK1-S1 |
| --- | --- | --- | --- | --- |
| Raw reads | 52,331,777 | 57,073,058 | 52,288,750 | 48,639,703 |
| Mapped reads | 37,736,940 | 38,232,758 | 42,559,755 | 36,841,966 |
| Unmapped reads | 14,011,504 | 18,099,900 | 8,840,548 | 11,057,436 |
| Multi-Hit reads | 583,333 | 740,400 | 888,447 | 740,301 |
| Average map rate (%) | 72.1 | 67.0 | 81.4 | 75.7 |
| Average map rate after the coverage cutoff | 71.9 | 66.7 | 80.1 | 75.5 |
