# Supplementary material for: Global DNA Methylation in the Chestnut Blight Fungus Cryphonectria parasitica and Genome-Wide Changes in DNA Methylation Accompanied with Sectorization
Source: Front Plant Sci. 2018 Feb 2;9:103. doi: 10.3389/fpls.2018.00103 (PMC5801561; doi:10.3389/fpls.2018.00103)
Supplement: Supplementary file 3 [file Table_3.DOCX]

**Supplemental Table S3.** List of loci that are validated using BS-PCR

| Locus | | Strain | W^a^ | B^b^ | W∩B^c^ |
| --- | --- | --- | --- | --- | --- |
| Scaffold | Position |  |  |  |  |
| 13 | 32825-32935 | EP155/2 | 11 | 9 | 9 |
|  |  | TdBCK1 | 11 | 9 | 9 |
|  |  | TdBCK1-S1 | 11 | 11 | 11 |
|  |  | TcBCK1-S1 | 11 | 9 | 9 |
|  |  | *∆CpDmt1* | NS^d^ | 3 | - |
|  |  | *∆CpDmt2* | NS | 0 | - |
| 13 | 33015-33109 | EP155/2 | 6 | 6 | 5 |
|  |  | TdBCK1 | 5 | 7 | 5 |
|  |  | TdBCK1-S1 | 7 | 7 | 6 |
|  |  | TcBCK1-S1 | 6 | 7 | 5 |
|  |  | *∆CpDmt1* | NS | 3 | - |
|  |  | *∆CpDmt2* | NS | 0 | - |

^a^Number of mC sites in WGBS data.

^b^Number of mC sites in BS-PCR experiment.

^c^Number of mC sites that overlap between WGBS and BS-PCR.

^d^NS, not sequenced for WGBS in this experiment.
