# Supplementary material for: Global DNA Methylation in the Chestnut Blight Fungus Cryphonectria parasitica and Genome-Wide Changes in DNA Methylation Accompanied with Sectorization
Source: Front Plant Sci. 2018 Feb 2;9:103. doi: 10.3389/fpls.2018.00103 (PMC5801561; doi:10.3389/fpls.2018.00103)
Supplement: Supplementary file 4 [file Table_4.DOCX]

**Supplemental Table S4.** Summary of global mC data for each strain

| EP155/2 | |  |  |  |
| --- | --- | --- | --- | --- |
| Context | Methylated  read | Unmethylated  read | Methylation ratio  (a/a+b)×100 | Number of mC sites  (%)^a^ |
| C | 104,353,301 | 2,568,282,094 | 3.90 | 1,410,293 (100%) |
| CHG | 37,894,269 | 836,416,917 | 4.33 | 306,837 (21.8%) |
| CHH | 66,239,878 | 1,493,360,457 | 4.25 | 1,009,758 (71.6%) |
| CpG | 219,154 | 238,504,720 | 0.09 | 93,698 (6.6%) |
| TdBCK1 | |  |  |  |
| Context | Methylated  read | Unmethylated  read | Methylation ratio  (a/a+b)×100 | Number of mC sites  (%)^a^ |
| C | 71,711,693 | 2,670,102,182 | 2.62 | 1,694,399 (100%) |
| CHG | 25,312,356 | 900,820,836 | 2.73 | 382,817 (22.6%) |
| CHH | 46,210,619 | 1,501,950,808 | 2.98 | 1,200,660 (70.9%) |
| CpG | 188,718 | 267,330,538 | 0.07 | 110,922 (6.5%) |
| TdBCK1-S1 | |  |  |  |
| Context | Methylated  read | Unmethylated  read | Methylation ratio  (a/a+b)×100 | Number of mC sites  (%)^a^ |
| C | 36,622,678 | 3,687,246,174 | 0.98 | 1,575,942 (100%) |
| CHG | 13,359,153 | 1,252,694,430 | 1.06 | 352,889 (22.4%) |
| CHH | 23,049,538 | 2,023,990,100 | 1.13 | 1,082,083 (68.7%) |
| CpG | 213,987 | 410,561,644 | 0.05 | 140,970 (8.9%) |
| TcBCK1-S1 | |  |  |  |
| Context | Methylated  read | Unmethylated  read | Methylation ratio  (a/a+b)×100 | Number of mC sites  (%)^a^ |
| C | 62,717,567 | 2,907,734,272 | 2.11 | 1,576,653 (100%) |
| CHG | 23,308,122 | 994,084,230 | 2.29 | 351,068 (22.3%) |
| CHH | 39,215,041 | 1,597,202,078 | 2.40 | 1,104,315 (70.1%) |
| CpG | 194,404 | 316,447,964 | 0.06 | 120,970 (7.7%) |

Strains are indicated at the top of the corresponding table.

*Methylation ratio: methylated read/(methylated read+unmethylated read)×100.

*EP155/2 > TdBCK1 > TcBCK1-S1 > TdBCK1-S1.

^a^Proportion of the different DNA context within the whole mC sites.
