# Supplementary material for: Global DNA Methylation in the Chestnut Blight Fungus Cryphonectria parasitica and Genome-Wide Changes in DNA Methylation Accompanied with Sectorization
Source: Front Plant Sci. 2018 Feb 2;9:103. doi: 10.3389/fpls.2018.00103 (PMC5801561; doi:10.3389/fpls.2018.00103)
Supplement: Supplementary file 5 [file Table_5.DOCX]

**Supplemental Table S5.** The list of predicted genes found in specific domain of supercontig 4

| Genomic position | Locus | Strand | Length | Protein Domain^*^ | Gene Ontology |
| --- | --- | --- | --- | --- | --- |
| scaffold_4:1527819-1530841 | Crypa1.estExt_fgenesh1_pm.C_20251 | + | 778 | IPR004813  IPR004648 | GO:0055085  transmembrane transport |
| scaffold_4:1532406-1533150 | estExt_fgenesh1_kg.C_40176 | - | 79 | None |  |
| scaffold_4:1533156-1533950 | Crypa1.estExt_fgenesh1_pm.C_20253 | + | 79 | None |  |
| scaffold_4:1536598-1537250 | Crypa1.fgenesh1_pg.C_scaffold_2000369 | - | 162 | None |  |

^*^ Protein domains were predicted by InterPro Scan (https://www.ebi.ac.uk/interpro/sequence-search). InterPro accessions definitions: IPR004813 (Oligopeptide transporter, OPT superfamily), IPR004648 (Tetrapeptide transporter, OPT1/isp4), IPR012334 (Pectin lyase fold), IPR011050 (Pectin lyase fold/virulence factor), and IPR000743 (Glycoside hydrolase, family 28).
