# Supplementary material for: Global DNA Methylation in the Chestnut Blight Fungus Cryphonectria parasitica and Genome-Wide Changes in DNA Methylation Accompanied with Sectorization
Source: Front Plant Sci. 2018 Feb 2;9:103. doi: 10.3389/fpls.2018.00103 (PMC5801561; doi:10.3389/fpls.2018.00103)
Supplement: Supplementary file 6 [file Table_6.DOCX]

**Supplemental Table S6.** Distribution of mC site in different genomic features

| Type | EP155/2 | TdBCK1 | TdBCK1-S1 | TcBCK1-S1 |
| --- | --- | --- | --- | --- |
| Upstream (1.5 kb) | 168,757 | 244,448 | 201,846 | 199,567 |
| Exon | 102,578 | 175,377 | 237,662 | 181,193 |
| Intron | 41,205 | 55,430 | 51,951 | 49,835 |
| Downstream (1.0 kb) | 110,916 | 167,007 | 126,089 | 127,021 |
| Intergenic region | 986,837 | 1,052,137 | 958,394 | 1,018,737 |
| Total of mC site | 1,410,293 | 1,694,399 | 1,575,942 | 1,576,353 |
