# Supplementary material for: Global DNA Methylation in the Chestnut Blight Fungus Cryphonectria parasitica and Genome-Wide Changes in DNA Methylation Accompanied with Sectorization
Source: Front Plant Sci. 2018 Feb 2;9:103. doi: 10.3389/fpls.2018.00103 (PMC5801561; doi:10.3389/fpls.2018.00103)
Supplement: Supplementary file 7 [file Table_7.DOCX]

**Supplemental Table S7.** DNA methyltransferase proteins in the fungal genomes

| UniProtKB | Organism | Gene description |
| --- | --- | --- |
| E9DT40 | *Metarhizium acridum* CQMa 102 (strain: CQMa 102) | Cytosine C5-DNA methyltransferase, putative |
| Q2KFY7 | *Magnaporthe oryzae* (strain 70-15 / ATCC MYA-4617 / FGSC 8958) (Rice blast fungus) | Putative uncharacterized protein |
| B0B065 | *Sordaria macrospora* | Putative DNA cytosine methyltransferase |
| Q8NJW0 | *Neurospora crassa* | RIP defective |
| B2AUK7 | *Podospora anserina* (strain S / ATCC MYA-4624 / DSM 980 / FGSC 10383) (Pleurage anserina) | Podospora anserina S mat+ genomic DNA chromosome 1, supercontig 4 |
| G2XR28 | *Botryotinia fuckeliana* (strain T4) | Uncharacterized protein |
| A7EIB2 | *Sclerotinia sclerotiorum* (strain ATCC 18683 / 1980 / Ss-1) | Putative uncharacterized protein |
| A7E505 | *Sclerotinia sclerotiorum* (strain ATCC 18683 / 1980 / Ss-1) | Putative uncharacterized protein |
| G2YJ06 | *Botryotinia fuckeliana* (strain T4) | Uncharacterized protein |
| E3S1K2 | *Pyrenophora teres f. teres* (strain 0-1) | Putative uncharacterized protein |
| E4ZP97 | *Leptosphaeria maculans* (strain JN3 / isolate v23.1.3 / race Av1-4-5-6-7-8) | Putative uncharacterized protein |
| A2R4V8 | *Aspergillus niger* (strain CBS 513.88 / FGSC A1513) | Aspergillus niger contig An15c0090, genomic contig |
| C1H1B8 | *Paracoccidioides lutzii* (strain ATCC MYA-826 / Pb01) | C-5 cytosine methyltransferase DmtA |
| C5JRX9 | *Ajellomyces dermatitidis* (strain SLH14081) | C-5 cytosine methyltransferase DmtA |
| D5GQ57 | *Tuber melanosporum* (strain Mel28) | Uncharacterized protein |
| O13369 | *Ascobolus immersus* | Masc1 |
| U5HAC8 | *Microbotryum violaceum* (strain p1A1 Lamole) | Uncharacterized protein |
| F4RK98 | *Melampsora larici-populina* (strain 98AG31 / pathotype 3-4-7) | Putative uncharacterized protein |
| E3K0G1 | *Puccinia graminis f. sp. tritici* (strain CRL 75-36-700-3 / race SCCL) | Putative uncharacterized protein |
| F8PG37 | *Serpula lacrymans* var. *lacrymans* (strain S7.3) | Putative uncharacterized protein |
| B8PG48 | *Postia placenta* (strain ATCC 44394 / Madison 698-R) | Predicted protein (Fragment) |
| B0CNH7 | *Laccaria bicolor* (strain S238N-H82 / ATCC MYA-4686) | Predicted protein |
| A8N3W7 | *Coprinopsis cinerea* (strain Okayama-7 / 130 / ATCC MYA-4618 / FGSC 9003) | Uncharacterized protein |
| O42731 | *Ascobolus immersus* | Cytosine-specific methyltransferase |
| F8Q7P9 | *Serpula lacrymans* var. *lacrymans* (strain S7.3) | Cytosine-specific methyltransferase |
| B0CSP1 | *Laccaria bicolor* (strain S238N-H82 / ATCC MYA-4686) | Cytosine-specific methyltransferase |
| D8PV89 | *Schizophyllum commune* (strain H4-8 / FGSC 9210) | Cytosine-specific methyltransferase |
| A8NEZ8 | *Coprinopsis cinerea* (strain Okayama-7 / 130 / ATCC MYA-4618 / FGSC 9003) | Cytosine-specific methyltransferase |
| B0B066 | *Sordaria macrospora* | Putative DNA cytosine methyltransferase |
| Q96W73 | *Neurospora crassa* | DNA methyltransferase Dim-2 |
| B2AKW2 | *Podospora anserina* (strain S / ATCC MYA-4624 / DSM 980 / FGSC 10383) | Podospora anserina S mat+ genomic DNA chromosome 5, supercontig 9 |
| G4NDQ4 | *Magnaporthe oryzae* (strain 70-15 / ATCC MYA-4617 / FGSC 8958) | Modification methylase DdeI |
| E9DV68 | *Metarhizium acridum* (strain CQMa 102) | Cytosine-specific methyltransferase |
| G2YFT1 | *Botryotinia fuckeliana* (strain T4) | Cytosine-specific methyltransferase |
| A7ERM2 | *Sclerotinia sclerotiorum* (strain ATCC 18683 / 1980 / Ss-1) | Cytosine-specific methyltransferase |
| D5G9M5 | *Tuber melanosporum* (strain Mel28) | Uncharacterized protein |
| C1H2T7 | *Paracoccidioides lutzii* (strain ATCC MYA-826 / Pb01) | DNA methyltransferase Dim-2 |
| C5JDC8 | *Ajellomyces dermatitidis* (strain SLH14081) | DNA methyltransferase Dim-2 |
| E3RWK9 | *Pyrenophora teres f. teres* (strain 0-1) | Putative uncharacterized protein |
| E4ZS83 | *Leptosphaeria maculans* (strain JN3 / isolate v23.1.3 / race Av1-4-5-6-7-8) | Putative uncharacterized protein |
| P40999 | *Schizosaccharomyces pombe* (strain 972 / ATCC 24843) | tRNA (cytosine(38)-C(5))-methyltransferase |
