# Supplementary material for: Global DNA Methylation in the Chestnut Blight Fungus Cryphonectria parasitica and Genome-Wide Changes in DNA Methylation Accompanied with Sectorization
Source: Front Plant Sci. 2018 Feb 2;9:103. doi: 10.3389/fpls.2018.00103 (PMC5801561; doi:10.3389/fpls.2018.00103)
Supplement: Supplementary file 8 [file Image_1.PDF]

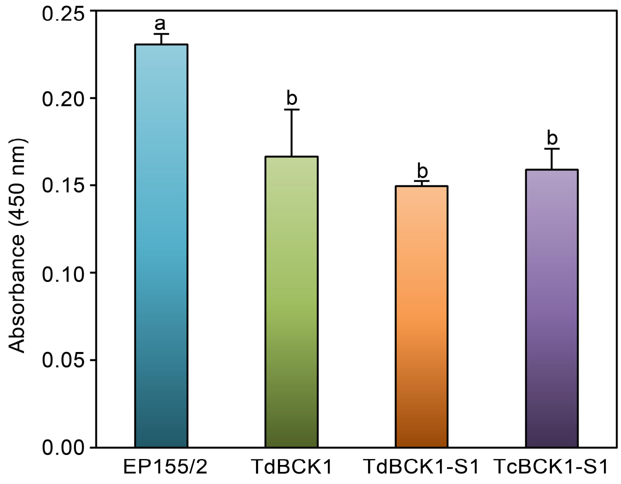

Supplemental Figure S1. An ELISA-like assay using the mC-specific antibody was used to quantify methylated DNA. At least three biological replicates for each strain were used. Different letters indicate significant differences between strains according to Duncan's multiple range test at $p = 0.05$.
