# Supplementary material for: Global DNA Methylation in the Chestnut Blight Fungus Cryphonectria parasitica and Genome-Wide Changes in DNA Methylation Accompanied with Sectorization
Source: Front Plant Sci. 2018 Feb 2;9:103. doi: 10.3389/fpls.2018.00103 (PMC5801561; doi:10.3389/fpls.2018.00103)
Supplement: Supplementary file 9 [file Image_2.PDF]

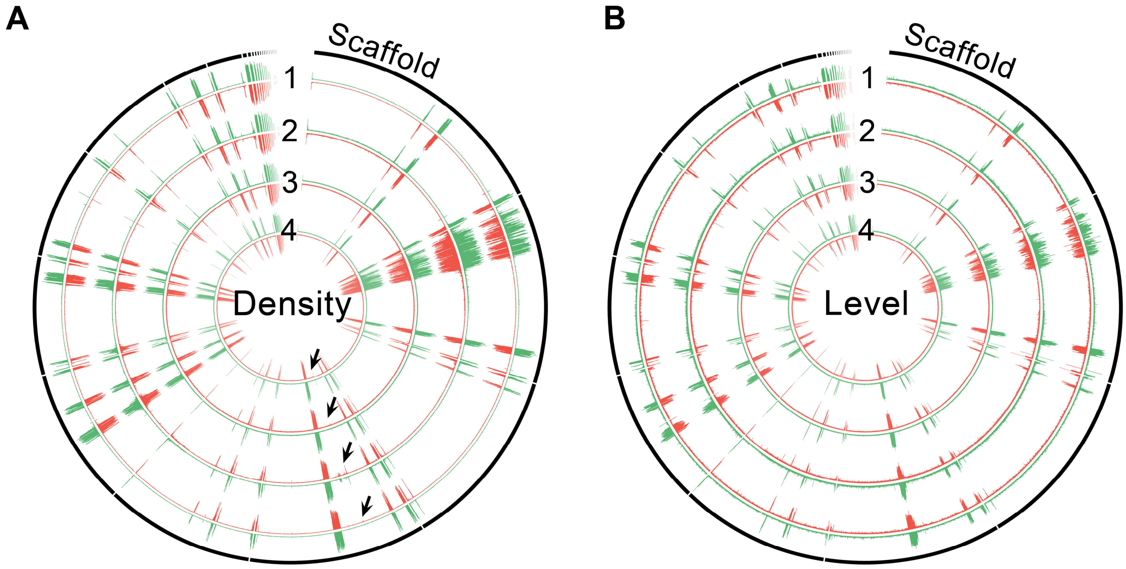

Supplemental Figure S2. Chromosomal distribution of DNA methylation in the genome of *C. parasitica.* **(A)** The distribution of DNA methylation is represented by the methylation density (number of mC sites/W, where W is 10 kb). Strains are indicated by numbers: 1-4 represent EP155/2, TdBCK1, TdBCK1-S1, and TcBCK1-S1, respectively. Scaffolds (supercontigs) are represented by the outer black circles in order of scaffold number. Green and red bars indicate methylation in Watson and Crick strands, respectively. Note that an arrow indicates an occurrence of new methylated domain in–supercontig 4 of the mutant strain. **(B)** The distribution of DNA methylation is represented by the methylation level (number of mC reads/number of total reads). Strains, scaffolds, and DNA strands are identified as Supplementary Figure S2A.
