# Supplementary material for: Global DNA Methylation in the Chestnut Blight Fungus Cryphonectria parasitica and Genome-Wide Changes in DNA Methylation Accompanied with Sectorization
Source: Front Plant Sci. 2018 Feb 2;9:103. doi: 10.3389/fpls.2018.00103 (PMC5801561; doi:10.3389/fpls.2018.00103)
Supplement: Supplementary file 10 [file Image_3.PDF]

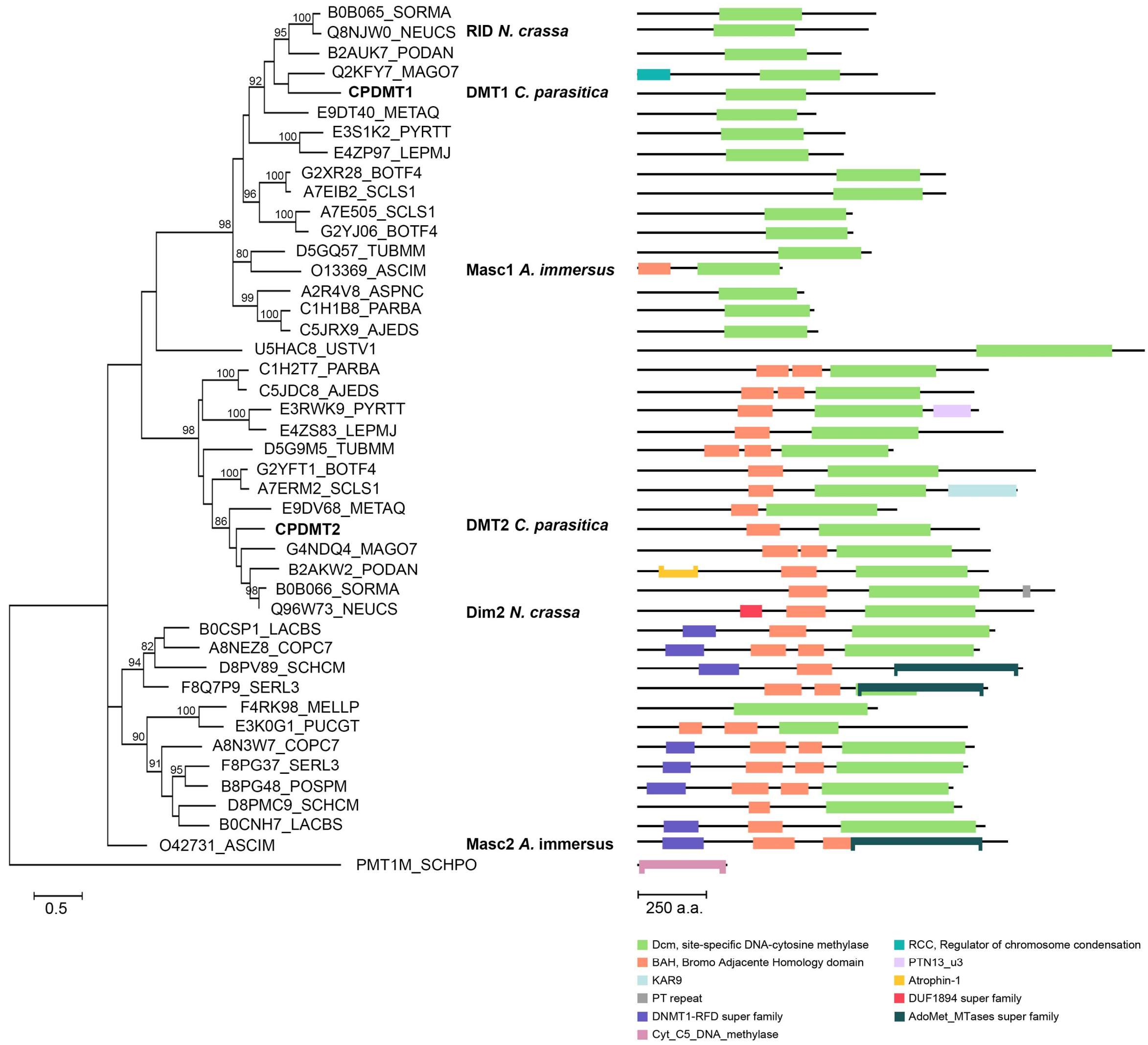

Supplemental Figure S3. Phylogenetic analysis of the deduced amino acid sequences of CpDMT1 and CpDMT2. Domain architecture is presented in the parallel panel. A list of organism information for GenBank accession numbers is provided in Supplementary Table S7.
