# Supplementary material for: Global DNA Methylation in the Chestnut Blight Fungus Cryphonectria parasitica and Genome-Wide Changes in DNA Methylation Accompanied with Sectorization
Source: Front Plant Sci. 2018 Feb 2;9:103. doi: 10.3389/fpls.2018.00103 (PMC5801561; doi:10.3389/fpls.2018.00103)
Supplement: Supplementary file 11 [file Image_4.PDF]

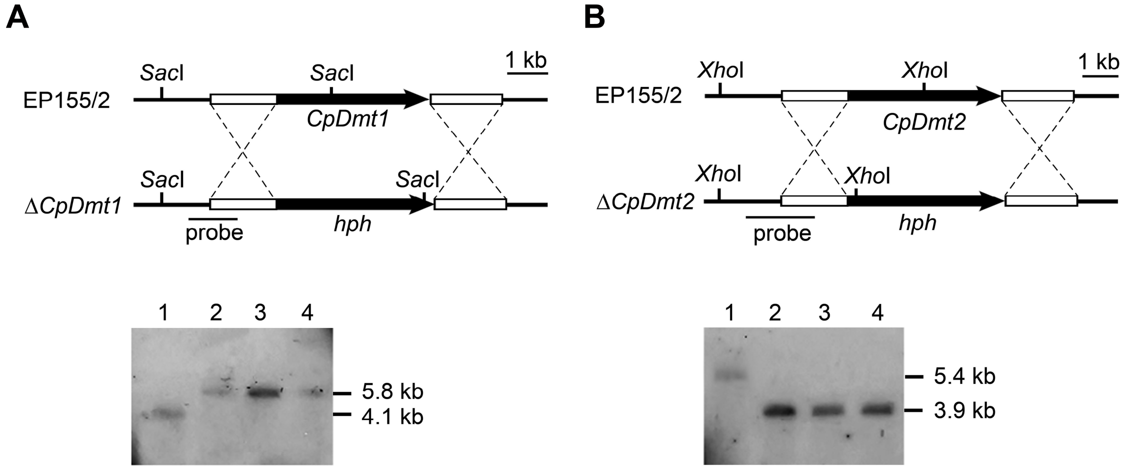

Supplemental Figure S4. Restriction map and Southern blot analyses of *CpDmt1*-null mutant **(A)** and *CpDmt2*-null mutant **(B)** strains. Restriction maps of the *CpDmt1* and *CpDmt2* genomic regions and the expected gene replacement are shown under the wild-type alleles. Flanking regions and expected replacement ORF regions are indicated by open and black boxes, respectively. *hph* represents the hygromycin B resistance cassette. Genes outside the replacement vector are indicated by lines. Southern blot analyses of *SacI*- and *Xho*I-digested DNA from the wild-type EP155/2 strain and three single-spored transformants of the *CpDmt1*- and *CpDmt2*-null mutants, respectively, were conducted.
